# Supplementary material for: Nonlocal pseudopotential energy density functional for orbital-free density functional theory
Source: Nat Commun. 2022 Mar 16;13:1385. doi: 10.1038/s41467-022-29002-3 (PMC8927098; doi:10.1038/s41467-022-29002-3)
Supplement: Supplementary file 1 — Supplementary Information [file 41467_2022_29002_MOESM1_ESM.pdf]

Supplementary Information

Nonlocal Pseudopotential Energy Density Functional for Orbital-Free Density Functional Theory

Q. X. et al.

Supplementary Notes

An explicitly density-dependent modified Gaussian density matrix functional [see Eqs. (6)-(8) in the maintext] for the NLPPF scheme was obtained by using the approximate kinetic energy density (KED). Here, we provide the details of two KEDs derived from the integrands of Wang-Teter (WT)[1] and Xu-Wang-Ma (XWM)[2] KEDFs directly:

$$t_s^{WT/XWM}[\rho](\mathbf{r}) = t^{TF}[\rho](\mathbf{r}) + t^{vW}[\rho](\mathbf{r}) + t_{nl}^{WT/XWM}[\rho](\mathbf{r}), \tag{1}$$

where three terms on the right hand side of Eq. (1) are the KEDs of Thomas-Fermi[3–5] $t^{TF}[\rho](\mathbf{r}) = \frac{3}{10}(3\pi^2)^{2/3}\rho^{5/3}(\mathbf{r})$, von Weizsäcker[6] $t^{vW}[\rho](\mathbf{r}) = \frac{1}{8}\frac{|\nabla\rho(\mathbf{r})|^2}{\rho(\mathbf{r})}$, and the nonlocal term of WT/XWM functionals, respectively. The nonlocal KED of WT is given by

$$t_{nl}^{WT}[\rho](\mathbf{r}) \equiv \rho^{5/6}(\mathbf{r}) \int \omega_{WT}(\rho_0;\mathbf{r},\mathbf{r}')\rho^{5/6}(\mathbf{r}')d^3\mathbf{r}', \tag{2}$$

where $\omega_{WT}(\rho_0;\mathbf{r},\mathbf{r}')$ is the kernel of WT KEDF[1, 2, 7] and ρ_0 is the average electron density of the unit cell. For XWM, the nonlocal term of XWM KEDF can be written in a compact form:

$$\begin{aligned} T_{nl}^{XWM}[\rho] &= \int\int \rho^{\kappa+5/6}(\mathbf{r})\omega_1(\rho^*,\rho_0;\mathbf{r},\mathbf{r}')\rho^{\kappa+5/6}(\mathbf{r}')d^3r d^3r' \\ &+ \int\int \rho^{\kappa+5/6}(\mathbf{r})\omega_2(\rho_0;\mathbf{r},\mathbf{r}')\rho^{\kappa+11/6}(\mathbf{r}')d^3\mathbf{r}d^3\mathbf{r}', \end{aligned} \tag{3}$$

where $\omega_1(\rho^*,\rho_0;\mathbf{r},\mathbf{r}') = \frac{18G_{Lind}(\rho^*;|\mathbf{r}-\mathbf{r}'|)}{(6\kappa+5)^2\rho_0^{2\kappa}} - \frac{\rho^* G'(\mathbf{r},\mathbf{r}')}{(\kappa+5/6)^2\rho_0^{2\kappa}}$ and $\omega_2(\rho_0;\mathbf{r},\mathbf{r}') = \frac{G'(\mathbf{r},\mathbf{r}')}{[(\kappa+5/6)(\kappa+11/6)]\rho_0^{2\kappa}}$. $G_{Lind}(\rho^*,|\mathbf{r}-\mathbf{r}'|)$ and $G'(\mathbf{r},\mathbf{r}')$ were defined in Ref.[2]. ρ^* and κ are two adjustable parameters. Note that the second term in the right hand side of Eq. (3) causes the multiple definitions of XWM KED, such as:

$$\begin{aligned} t_{nl,\lambda}^{XWM}[\rho](\mathbf{r}) \equiv & \quad \rho^{\kappa+5/6}(\mathbf{r}) \int \omega_1(\rho^*,\rho_0;\mathbf{r},\mathbf{r}')\rho^{\kappa+5/6}(\mathbf{r}')d^3\mathbf{r}' \\ &+ \lambda \cdot \rho^{\kappa+5/6}(\mathbf{r}) \int \omega_2(\rho_0;\mathbf{r},\mathbf{r}')\rho^{\kappa+11/6}(\mathbf{r}')d^3\mathbf{r}' \\ &+ (1-\lambda) \cdot \rho^{\kappa+11/6}(\mathbf{r}) \int \omega_2(\rho_0;\mathbf{r},\mathbf{r}')\rho^{\kappa+5/6}(\mathbf{r}')d^3\mathbf{r}'. \end{aligned} \tag{4}$$

where λ is the linear combination coefficient. The parameters of $\kappa = 0$, $\rho^* = \rho_0$ and $\lambda = \frac{1}{2}$

are used as the default setting unless otherwise specified.

The Troullier-Martins nonlocal pseudopotentials (NLPPs)[8] are generated by the FHI98PP[9] code for all considered systems [see Supplementary Table **1**] and the p-channel of the NLPPs is used as the local pseudopotential of $V_{loc}(r)$ in OF-DFT. KS-DFT calculations using the projector augmented-wave (PAW)[10] and NLPP are performed by VASP[11, 12] and ARES packages[13], respectively. The k-point meshes are generated using the Monkhorst-Pack method[14] with the k-spacing of 0.10 Å^{-1}. The kinetic energy cutoff is 500 eV for all the simulations using VASP. The OF-DFT calculations are carried out by ATLAS[15, 16] using WT[1] and XWM[2] as KEDFs, and the corresponding KEDs are used to construct the NLPPFs. The generalized gradient approximation with the form of Perdew-Burke-Ernzerhof[17] is employed for both OF-DFT and KS-DFT calculations. The grid spacings of 0.18, 0.18, 0.22, 0.10, 0.15, 0.22, 0.12 and 0.15 Å are used in ATLAS/ARES for Li, Mg, Cs, Be, Cd, K, Zn and Li-Mg alloy, respectively. The parameters of A and q in NLPPFs are presented in Supplementary Table **2** carefully tuned to yield the bulk properties, which agree with the KS-DFT (NLPPs) predictions.

We performed the OF-DFT and KS-DFT calculations of Li, Mg, Cs, K and Zn structures including hexagonal-close-packed (HCP), face-centered cubic (FCC), body-centered cubic (BCC), simple cubic (SC), and cubic diamond (CD). For each structure, the bulk properties including the equilibrium cell volume (V_0), bulk modulus (B_0) were determined by fitting the energy versus volume curve against Murnaghan's equation of state[18]. Supplementary Table **3** lists the comparison of the results obtained by OF-DFT using the local pseudopotentials (e.g. BLPS[19] and OEPP[20]) and NLPPFs within WT/XWM against those from the KS-DFT calculations. Supplementary Table **4** lists the bulk properties of K and Zn calculated by both OF-DFT and KS-DSF.

The differences between WT-KED and KS-KED for Cd-SC are present in Figure **1**, which includes the systems with and without d-channel electrons. To assess the computational efficiency of the current scheme, the static simulations of the Cs-BCC supercells containing 128 to 16,000 atoms were performed by the ATLAS package for the OF-DFT calculations. Note that the one-orbital ensemble self-consistent field method[21] was employed to accelerate the total energy minimization for the OF-DFT within NLPPF scheme. For comparison, KS-DFT calculations are also included for the systems containing up to 256 atoms. All the calculations carried out using 2-node clusters, where each node contains two Intel(R)

Xeon(R) Gold 6240R CPUs (2.4 GHz base clock, 24 cores). The total wall time of the single point energy calculations versus the number of atoms curves were presented in Supplementary Figure **2**. It clearly shows that the computational costs of OF-DFT within both NLPSF and OEPP scale linearly with the number of atoms used in the simulation cell, in contrast to the cubic scaling KS-DFT approaches. Just as shown in Supplementary Figure. **2**, the linear scaling prefactor for OF-DFT using nonlocal pseudopotential is about 6 times larger than that using the local pseudopotential. This shows that the new OF-DFT framework still holds the potential applications for the simulation of large-scale systems containing millions of atoms.

To demonstrate the transferability of NLPPF, we have performed simulations of elemental systems (Li, Mg, K, and Cs), as well as Li-Mg alloy using NLPPF with WT[1] and XWM KEDFs[2]. The relative energies of these elemental systems and pair distribution functions of Li-Mg alloy are in reasonably good agreement with those of KS-PAW (Supplementary Figure **3** and Supplementary Figure **4**). Thus, we believe that NLPPF scheme is transferable to the other KEDFs[7, 22–24]. The further developments of NLPPF using the advanced KEDFs will be investigated in future.

Supplementary Table 1: The TM-NLPP parameters of the electron configuration, the core electron cutoff radius r_{nlc} and the pseudopotential cutoff radius r_c used in FHI98PP

Element	Configuration	r_{nlc}	$r_c(s/p)$
Li	$2s^1 2p^0$	1.80	2.40/2.40
K	$4s^1 4p^0$	3.20	3.25/3.17
Cs	$6s^1 6p^0$	3.50	4.00/4.40
Be	$2s^2 2p^0$	–	2.10/2.10
Mg	$3s^2 3p^0$	–	2.50/2.50
Zn	$4s^0 4p^2$	1.30	2.60/2.60
Cd	$5s^0 5p^2$	1.80	2.60/2.60

Supplementary Table 2: The parameters A, q and KED (as well as KEDF) used for OF-DFT calculations within NLPPF.

Element	KED	A	q
Li	WT	-0.851	0.999
	XWM	-0.908	1.155
K	WT	0.310	0.132
	XWM	0.139	-0.263
Cs	WT/XWM	0.590	0.095
Be	WT	-1.683	-0.005
Mg	WT	5.383	-2.140
	XWM	4.500	-1.512
Zn	WT	1.027	-0.185
Cd	WT	-0.655	-0.478

Supplementary Table 3: B_0 (GPa), E_R (eV/atom) and V_0 (Å^3/atom) for bulk Li, Mg, and Cs obtained by KS-DFT and OF-DFT. The bold texts highlight the results close to KS-DFT.

		Method	HCP	FCC	BCC	SC	CD
Li	B_0	KS (PAW)	13.9	13.6	13.9	12.1	5.3
		WT-BLPS	16.6	16.6	16.4	16.8	12.2
		WT-OEPP	15.4	15.4	15.5	**12.1**	6.4
		WT-NLPPF	**13.5**	**13.5**	**13.7**	11.0	5.4
		XWM-NLPPF	13.3	13.4	13.0	10.9	**5.3**
	V_0	KS-PAW	20.280	20.372	20.396	20.580	25.758
		WT-BLPS	19.308	19.294	**19.385**	19.945	22.462
		WT-OEPP	18.538	18.526	18.498	20.174	**26.803**
		WT-NLPPF	19.483	19.462	19.352	**20.844**	28.979
		XWM-NLPPF	**19.584**	**19.544**	19.328	21.570	28.098
	E_R	KS-PAW	0.000	0.000	0.001	**0.120**	0.515
		WT-BLPS	0.000	-0.001	0.002	0.137	**0.526**
		WT-OEPP	0.000	**0.000**	0.002	0.093	0.230
		WT-NLPPF	0.000	**0.000**	**0.001**	0.152	0.481
		XWM-NLPPF	0.000	**0.000**	0.000	0.164	0.480
Mg	B_0	KS-PAW	35.8	35.5	34.8	22.7	10.8
		WT-BLPS	**36.9**	**36.4**	**36.4**	**23.6**	11.4
		WT-OEPP	31.3	31.0	30.5	20.8	**10.4**
		WT-NLPPF	33.0	31.3	31.3	21.2	10.3
		XWM-NLPPF	31.9	31.2	31.8	20.5	11.7
	V_0	WT-PAW	22.838	23.071	22.826	27.478	40.242
		WT-BLPS	**23.052**	**23.227**	**23.003**	**27.196**	39.500
		WT-OEPP	24.276	24.459	24.358	28.937	41.207
		WT-NLPPF	23.194	23.924	23.730	28.274	40.770
		XWM-NLPPF	23.878	24.081	23.789	28.677	**40.624**
	E_R	KS-PAW	0.000	0.012	0.029	0.382	0.775
		WT-BLPS	0.000	**0.011**	**0.027**	**0.392**	0.840
		WT-OEPP	0.000	0.006	**0.027**	0.304	0.654
		WT-NLPPF	0.000	**0.011**	**0.031**	0.372	**0.783**
		XWM-NLPPF	0.000	0.016	0.021	0.350	0.746
Cs	B_0	KS-PAW	2.0	1.9	2.1	1.6	0.7
		WT-OEPP	2.5	2.5	2.6	2.1	**0.8**
		WT-NLPPF	**2.1**	**2.1**	2.2	**1.6**	**0.6**
		XWM-NLPPF	**2.1**	**2.1**	**2.1**	**1.6**	0.5
	V_0	KS-PAW	117.726	118.425	116.453	128.627	189.997
		WT-OEPP	107.533	107.420	107.028	112.485	153.853
		WT-NLPPF	**121.461**	**121.397**	**120.875**	127.935	180.329
		XWM-NLPPF	121.877	121.795	121.254	**129.026**	**186.649**
	E_R	KS-PAW	0.000	0.001	0.001	0.106	0.298
		WT-OEPP	0.000	-0.001	-0.001	**0.101**	0.315
		WT-NLPPF	0.000	**0.000**	-0.001	0.098	**0.288**
		XWM-NLPPF	0.000	-0.001	-0.001	0.098	0.285

Supplementary Table 4: B_0 (GPa), E_R (eV/atom) and V_0 (Å^3/atom) for bulk K and Zn obtained by KS-DFT and OF-DFT. The bold texts highlight the results close to KS-DFT.

Element	Properties	Method	HCP	FCC	BCC	SC	CD
K	B_0	KS-PAW	3.7	3.4	3.5	2.6	1.2
		WT-OEPP	3.8	3.8	3.8	3.0	1.3
		WT-NLPPF	3.6	**3.7**	**3.7**	**2.9**	**1.2**
		XWM-NLPPF	3.6	**3.7**	3.8	2.9	1.1
	V_0	KS-PAW	73.794	74.204	73.570	79.138	112.070
		WT-OEPP	71.342	71.287	71.087	75.262	102.808
		WT-NLPPF	**72.178**	**72.186**	**71.862**	76.585	106.231
		XWM-NLPPF	72.023	72.024	71.692	**76.837**	**106.677**
	E_R	KS-PAW	0.000	0.001	0.001	0.106	0.298
		WT-OEPP	0.000	0.000	0.000	0.112	0.348
		WT-NLPPF	0.000	0.000	0.000	**0.108**	**0.318**
		XWM-NLPPF	0.000	0.000	0.000	0.111	0.325
Zn	B_0	KS-PAW	71.5	67.3	63.4	46.8	25.4
		KS-NLPP	83.6	81.9	79.9	49.8	24.3
		WT-OEPP	97.9	94.7	94.9	56.4	30.9
		WT-NLPPF	78.1	77.6	76.7	49.3	27.0
	V_0	KS-PAW	15.335	15.236	15.435	18.313	24.821
		KS-NLPP	12.040	12.238	12.066	14.692	21.469
		WT-OEPP	10.956	11.178	11.036	13.557	20.040
		WT-NLPPF	12.424	12.587	12.472	14.959	21.394
	E_R	KS-PAW	0.000	0.021	0.082	0.224	0.466
		KS-NLPP	0.000	0.027	0.051	0.463	0.907
		WT-OEPP	0.000	0.042	0.055	0.563	1.096
		WT-NLPPF	0.000	0.026	0.045	0.434	0.888

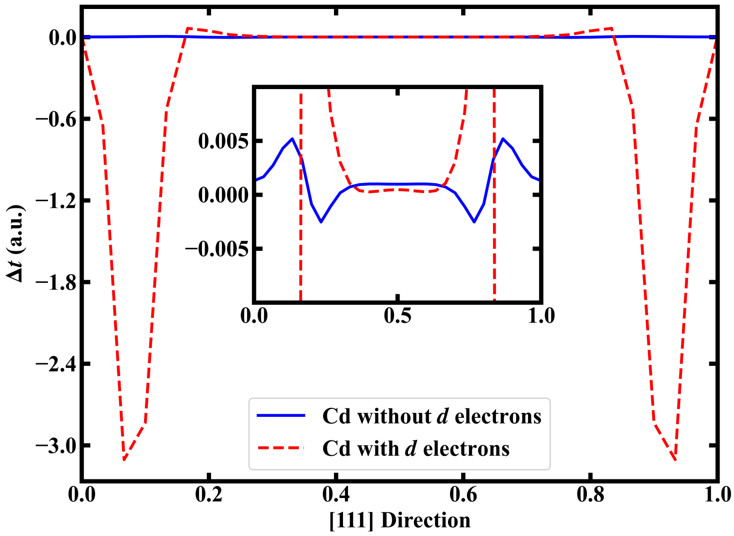

Supplementary Figure 1: KED differences along [111] direction for Cd-SC structure. The differences between WT-KED and KS-KED are calculated by $\Delta t(\mathbf{r}) \equiv t_s^{WT}[\rho^{KS}](\mathbf{r}) - t_s^{KS}(\mathbf{r})$.

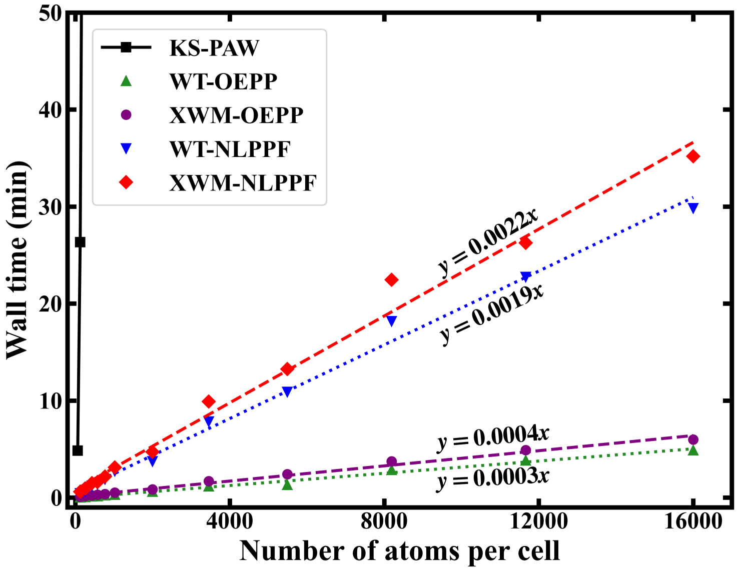

Supplementary Figure 2: The wall times for the single-point calculations of Cs-BCC supercell structures. The simulations of Cs-BCC supercells containing 128 to 16,000 atoms were performed by OF-DFT using NLPPF and OEPP in comparison with KS-DFT using PAW.

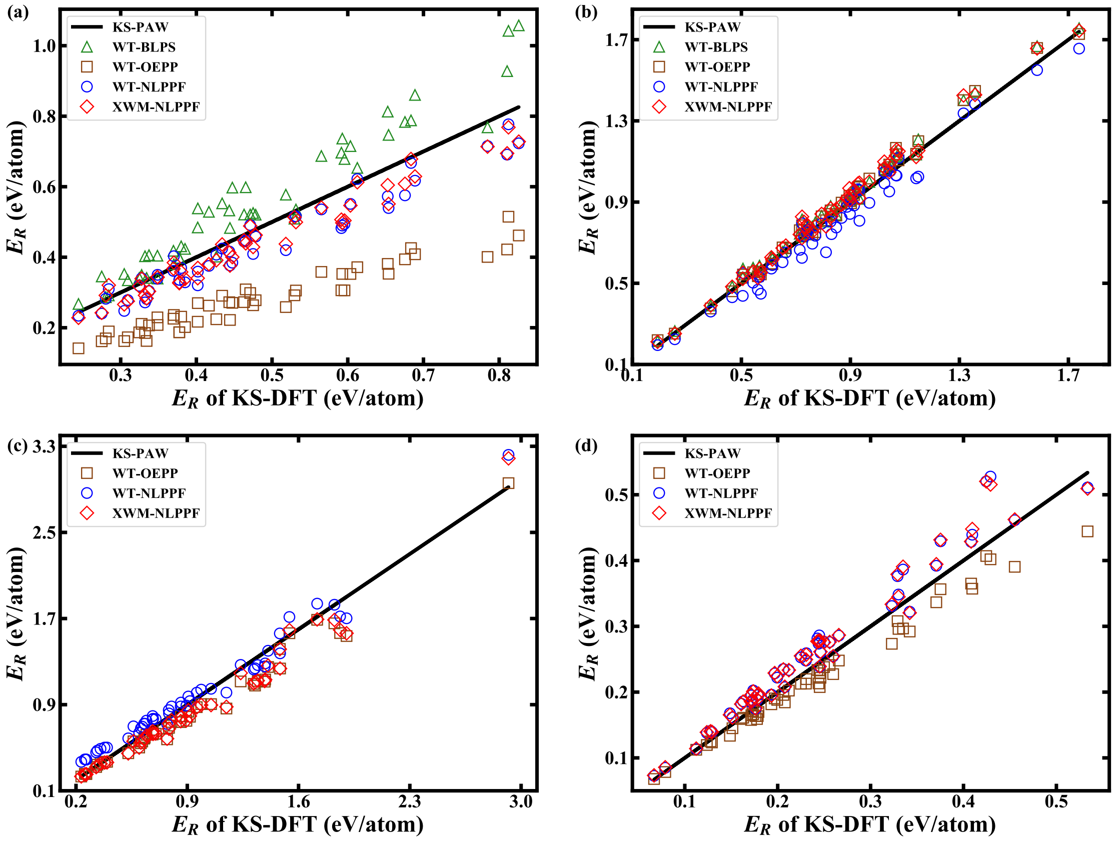

Supplementary Figure 3: **Relative energies of random structures.** For each system, 50 random structures are generated by CALYPSO software[25, 26]. The relative energies of (a) Li, (b) Mg, (c) K, and (d) Cs random structures obtained by OF-DFT using BLPS, OEPP and NLPPF in comparison with KS-DFT using the PAW method.

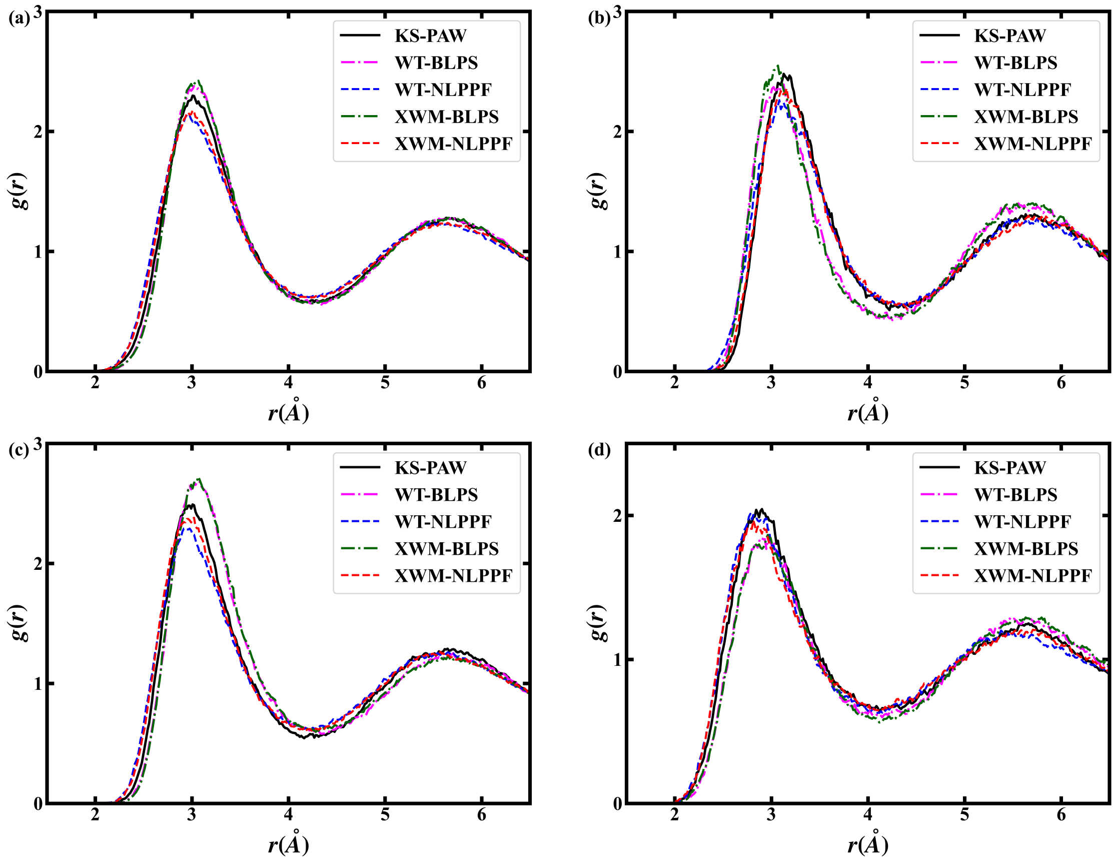

Supplementary Figure 4: **Pair distribution functions for $Li_{54}Mg_{54}$ alloy.** The molecular dynamic simulations of Li-Mg alloy are perform in the canonical ensemble at 1000 K applying the Nosé-Hoover thermostat[27, 28] simulations. (a) Total, (b) Mg-Mg, (c) Li-Mg, and (d) Li-Li pair distribution functions.

Supplementary References

[1] L.-W. Wang and M. P. Teter, Physical Review B **45**, 13196 (1992).
[2] Q. Xu, Y. Wang, and Y. Ma, Physical Review B **100**, 205132 (2019).
[3] L. H. Thomas, Mathematical Proceedings of the Cambridge Philosophical Society **23**, 542–548 (1927).
[4] E. Fermi, Rend. Accad. Naz. Lincei **6**, 5 (1927).
[5] E. Fermi, Zeitschrift für Physik **48**, 73 (1928).
[6] C. v. Weizsäcker, Zeitschrift für Physik **96**, 431 (1935).
[7] Y. A. Wang, N. Govind, and E. A. Carter, Physical Review B **60**, 16350 (1999).
[8] N. Troullier and J. L. Martins, Physical Review B **43**, 1993 (1991).
[9] M. Fuchs and M. Scheffler, Computer Physics Communications **119**, 67 (1999).
[10] P. E. Blöchl, Physical Review B **50**, 17953 (1994).
[11] G. Kresse and J. Furthmüller, Physical Review B **54**, 11169 (1996).
[12] G. Kresse and J. Furthmüller, Computational Materials Science **6**, 15 (1996).
[13] Q. Xu, S. Wang, L. Xue, X. Shao, P. Gao, J. Lv, Y. Wang, and Y. Ma, Journal of Physics: Condensed Matter **31**, 455901 (2019).
[14] H. J. Monkhorst and J. D. Pack, Physical Review B **13**, 5188 (1976).
[15] W. Mi, X. Shao, C. Su, Y. Zhou, S. Zhang, Q. Li, H. Wang, L. Zhang, M. Miao, Y. Wang, et al., Computer Physics Communications **200**, 87 (2016).
[16] X. Shao, Q. Xu, S. Wang, J. Lv, Y. Wang, and Y. Ma, Computer Physics Communications **233**, 78 (2018).
[17] J. P. Perdew, K. Burke, and M. Ernzerhof, Physical Review Letters **77**, 3865 (1996).
[18] F. Murnaghan, J. Franklin Inst. **197**, 98 (1924).
[19] C. Huang and E. A. Carter, Physical Chemistry Chemical Physics **10**, 7109 (2008).
[20] W. Mi, S. Zhang, Y. Wang, Y. Ma, and M. Miao, The Journal of Chemical Physics **144**, 134108 (2016).
[21] X. Shao, W. Mi, and M. Pavanello, The Journal of Physical Chemistry Letters **12**, 4134 (2021).
[22] C. Huang and E. A. Carter, Physical Review B **81**, 045206 (2010).
[23] W. Mi and M. Pavanello, Physical Review B **100**, 041105 (2019).
[24] Q. Xu, J. Lv, Y. Wang, and Y. Ma, Physical Review B **101**, 045110 (2020).
[25] Y. Wang, J. Lv, L. Zhu, and Y. Ma, Physical Review B **82**, 094116 (2010).
[26] Y. Wang, J. Lv, L. Zhu, and Y. Ma, Computer Physics Communications **183**, 2063 (2012).
[27] S. Nosé, The Journal of Chemical Physics **81**, 511 (1984).
[28] W. G. Hoover, Physical Reveiew A **31**, 1695 (1985).
